# Ultrasonic liver steatosis quantification by a learning-based acoustic model from a novel shear wave sequence

**DOI:** 10.1186/s12938-019-0742-2

**Published:** 2019-12-21

**Authors:** Xiudong Shi, Wen Ye, Fengjun Liu, Rengyin Zhang, Qinguo Hou, Chunzi Shi, Jinhua Yu, Yuxin Shi

**Affiliations:** 1 Department of Radiology, Shanghai Public Health Clinical Center, Fudan University, Shanghai, China; 2 Department of Pathology, School of Basic Medical Science, Shanghai University of Traditional Chinese Medicine, Shanghai, China; 3 Department of Electronic Engineering, Fudan University, Shanghai, China; 4 Key Laboratory of Medical Imaging, Computing and Computer Assisted Intervention, Shanghai, China

**Keywords:** Liver steatosis quantification, Ultrasonic and shear wave parameter estimation, Learning-based model

## Abstract

**Background:**

An efficient and accurate approach to quantify the steatosis extent of liver is important for clinical practice. For the purpose, we propose a specific designed ultrasound shear wave sequence to estimate ultrasonic and shear wave physical parameters. The utilization of the estimated quantitative parameters is then studied.

**Results:**

Shear wave attenuation, shear wave absorption, elasticity, dispersion slope and echo attenuation were simultaneously estimated and quantified from the proposed novel shear wave sequence. Then, a regression tree model was utilized to learn the connection between the space represented by all the physical parameters and the liver fat proportion. MR mDIXON quantification was used as the ground truth for liver fat quantification. Our study included a total of 60 patients. Correlation coefficient (CC) with the ground truth were applied to mainly evaluate different methods for which the corresponding values were − 0.25, − 0.26, 0.028, 0.045, 0.46 and 0.83 for shear wave attenuation, shear wave absorption, elasticity, dispersion slope, echo attenuation and the learning-based model, respectively. The original parameters were extremely outperformed by the learning-based model for which the root mean square error for liver steatosis quantification is only 4.5% that is also state-of-the-art for ultrasound application in the related field.

**Conclusions:**

Although individual ultrasonic and shear wave parameters were not perfectly adequate for liver steatosis quantification, a promising result can be achieved by the proposed learning-based acoustic model based on them.

## Background

Liver steatosis is the buildup of triglycerides in the form of lipid droplets in liver which can be a result of several causes such as alcohol consumption, viral hepatitis or metabolic dysfunction [1, 2]. When the fat proportion of liver is larger than 5–10%, it is considered as fatty liver disease that with further progress associated with inflammation may irreversibly result in severe conditions such as hepatocellular carcinoma, diabetes mellitus or other metabolic complications [1, 3]. The prevalence of fatty liver disease is estimated as high as around 30% of the population [4]. An accurate and efficient method to diagnose liver fat extent is important for the clinical practice.

To quantitatively evaluate liver fat fraction nowadays, liver biopsy can be used which is essential for the diagnosis of non-alcoholic steatohepatitis (NASH) and is the only reliable procedure that differentiates non-alcoholic fatty liver (NAFL) from NASH, despite limitations due to sampling variability [5]. However, such invasive approach may encounter the problem of limited tissue sampling, serious complications and the low acceptance for patients [6]. Magnetic resonance imaging-based proton density fat fraction (MRI-PDFF) quantification is a procedure to non-invasively quantify liver fat extent by providing high-quality fat fraction maps of the entire liver. It is considered to be even more accurate than liver biopsy for the liver fat quantification [5]. MR mDIXON quantification is one of such technique specifically for Philips MRI system [7]. But the lack of accessibility due to the expensive cost of MRI systems significantly restricts the use of this technique in clinical practice. Medical ultrasound imaging is widely used and experienced doctors may qualitatively diagnose fatty liver disease based on ultrasound images. However, such diagnosis is subjective, operator-dependent and not quantitative [8].

Quantitative ultrasound demonstrates the capability to some extent for the diagnosis of fatty liver disease. The estimation of fundamental acoustic parameters such as echo attenuation and backscatter coefficient (BSC) were developed for liver fat quantification to characterize the tissue microstructure [9]. Some study has been published with a good result with a correlation between them and the result of MRI-PDFF as 0.79 [10]. Many previous studies also have evaluated the parameters such as echo attenuation, elasticity, viscosity (dispersion slope) and shear wave attenuation, respectively, for liver fat quantification [11–14]. However, to simultaneously evaluate different parameters and make the comparison for liver fat quantification, it is required to design a specific ultrasound transmitting and receiving sequence to realize the multiple parameter estimation simultaneously. One aim of our study is for this purpose. Meanwhile, we theoretically derive and further introduce the parameter estimation of shear wave absorption by the use of the designed ultrasound shear wave sequence. For all the estimated parameters, the performance of each one is evaluated on liver fat quantification with the results from MR mDIXON quantification as the ground truth. We further explores the use of the combination of all quantitative parameters on liver fat quantification by introducing a learning-based model, which achieves a significant improvement on performance and might provide a new direction for ultrasound tissue characterization in clinical application.

## Results

### Subjects

**Fig. 1:**
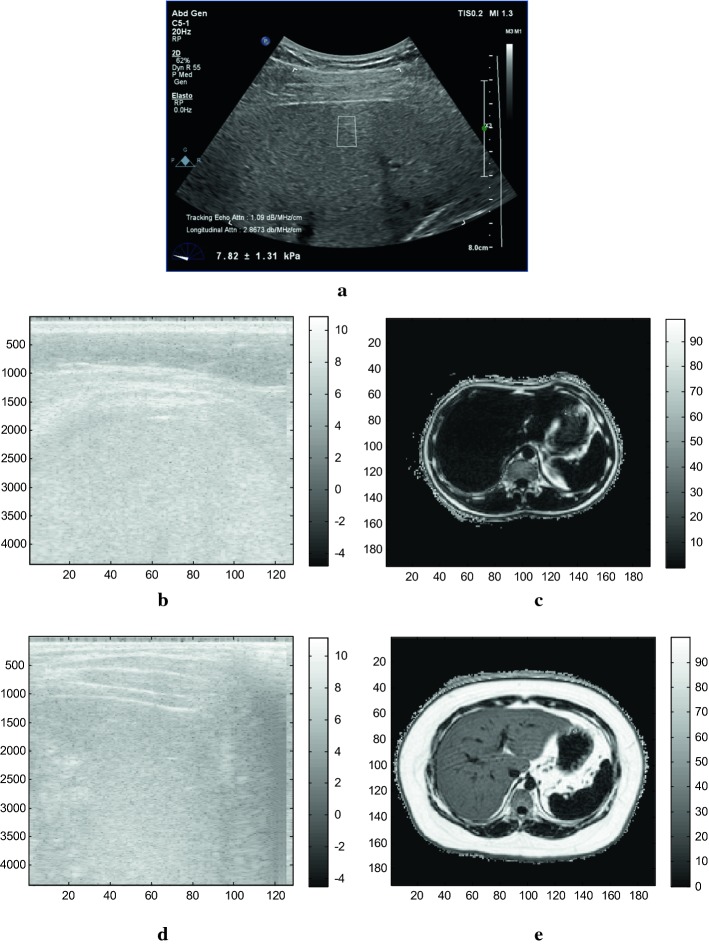
Demonstration of the acquired data in the study. **a** denotes the interface of the ultrasound data acquisition based on the designed ultrasound sequence. **b**, **d** The direct logarithmic results of the envelope of the corresponding raw radiofrequency ultrasound data. **c**, **e** The MRI fat quantification results. **b**, **c** are from the patient with liver fat fraction as 2.56%. **d**, **e** are from the patient with liver fat fraction as 29.45%. The scale bar in ultrasound images denotes the intensity and the one in MRI images denotes the percentage of fat fraction

The study included the patients who underwent routine ultrasound examination for the evaluation of the degree of hepatic steatosis from the physical examination center of Shanghai Public Health Clinical Center. Meanwhile, anthropometric measurement was performed and fasting venous blood samples were obtained for the determination of blood routine, liver function and hepatitis virus indication. Subjects were eligible for our study if they had no other known liver disease and did not have contraindications to MRI examination. Table 1 shows the complete inclusion and exclusion criteria [15] for which the assessment is mainly based on blood test and medical history of the patients. Based on these criteria, totally 60 participants who completed the ultrasound scanning for the data acquisition of multiple parameter estimation with the use of Philips EPIQ ultrasound system and the Philips MR mDIXON quantification examinations on the same day were selected for the study. An example is demonstrated for the acquired ultrasound and MR imaging data as in Fig. 1. The appropriate ultrasound imaging plane should mainly contain the liver parenchyma region at the depth from 3 to 5 cm by avoiding vessel or any other structural objective and the acquired ultrasound data for one patient should cover the patient’s liver parenchyma region as much as possible. The corresponding estimation results may thus represent the situation of the patient’s liver parenchyma region.

**Table 1:** Inclusion and exclusion criteria of patients for the study

Inclusion criteria
Age > 18 years
With a negative hepatitis B virus surface antigen and hepatitis C virus antibody
Willingness to undergo ultrasound and magnetic resonance examinations
Signed informed consent form
Exclusion criteria
With a history of other liver disease or diabetes
Excess alcoholic drinking [15] (≥ 20 g/day for men and ≥ 10 g/day for women)
Taking hypolipidemic drug, liver protectant, or drugs that could cause steatosis
With clinical symptoms or signs of other liver disease
MR examination contraindications (such as claustrophobia, cardiac pacemakers or metal implants)
Pregnant patients

The population of our study consisted of 22 women and 38 men for which the age ranged from 19 to 69 years old and the body mass index (BMI) ranged from 18.3 to 34.9 kg/m^2^. Hepatic fat proportion quantified by MR mDIXON quantification ranged from 2.0 to 47.2% with the average value as 9.1%. Table 2 shows the details of the related information. All patient data are available from the Department of Radiology in Shanghai Public Health Clinical Center affiliated Fudan University.

**Table 2:** Characteristics of the study patients

	MR mDIXON quantification hepatic fat content (%)				
	< 5	5–10	10–20	> 20	Total
Characteristic					
Number of participants	26	18	11	5	60
Age (year)	36 (23–67)	43 (19–69)	49 (27–68)	32 (26–42)	41 (19–69)
Sex (male/female)	20/6	9/9	5/6	4/1	38/22
Anthropometric measures					
Weight (kg)	64.9 ± 11.1	68.3 ± 8.1	73.7 ± 13.2	78.8 ± 11.9	68.8 ± 11.5
Height (cm)	168.5 ± 6.2	165.5 ± 5.4	164.3 ± 6.2	169.8 ± 5.2	167.0 ± 6.1
BMI (kg/m^2^)	22.9 ± 3.1	25.0 ± 2.4	27.2 ± 3.2	27.4 ± 2.8	24.7 ± 3.4
Ultrasound parameter estimated results					
Echo attenuation (dB/MHz/cm)	0.654 ± 0.132	0.713 ± 0.059	0.791 ± 0.117	0.832 ± 0.046	0.706 ± 0.121
Elasticity (kPa)	9.45 ± 3.90	9.75 ± 5.33	8.62 ± 2.41	10.3 ± 4.50	9.46 ± 4.15
Dispersion slope (m/s/Hz)	3.27 ± 10.2	5.54 ± 4.17	2.62 ± 10.1	2.97 ± 6.55	3.82 ± 8.48
Shear wave attenuation (Neper/m)	183.1 ± 57.9	178.7 ± 24.7	161.6 ± 41.3	138.7 ± 19.6	175.2 ± 46.3
Shear wave absorption (Neper/m)	61.1 ± 16.8	59.6 ± 11.3	55.3 ± 14.5	49.3 ± 9.55	58.9 ± 14.6
Model by using the combination of all parameters (%)	4.5 ± 4.3	7.8 ± 2.4	13.1 ± 4.3	25.9 ± 2.4	8.3 ± 6.7

### Phantom results

The sampling rate for the acquired ultrasound radiofrequency data is 40 MHz. The three push focus depths of the designed specific ultrasound shear wave sequence are 0.03 m, 0.04 m and 0.05 m, respectively. After engineer optimization, the spacing values used for the shear wave measurement points are correspondingly 0.0012 m, 0.0013 m and 0.0015 m.

Experiments on the 040GSE phantom (CIRS Inc., USA) were first performed to validate the parameter estimation methods. Table 3 shows the related results. The ground truth of echo attenuation was known for the phantom and it can be seen that the corresponding estimation results are well consistent with the ground truth. Meanwhile, the standard deviation of the results is small, which denotes the robustness of the echo attenuation estimation method and the signal stability of tracking echo. For the elasticity results, it can be seen that they are in the acceptable range of the designed 24 kPa for the Zerdine material of the CIRS phantom. Since there is no ground truth for the estimated parameters except echo attenuation, a house-made oil phantom was constructed to further evaluate the 040GSE phantom results. Compared with the 040GSE phantom, the house-made oil phantom was made with 20% gelatin and 20% animal oil (bovine) to simulate the situation with a relatively large extent of fat fraction. It can be seen that the dispersion is much larger than the one from the 040GSE phantom, which is as expected for a larger viscosity and validates the estimation algorithm to some extent. For the results of shear wave attenuation and shear wave absorption, their values change and manifest as negative correlation with the increase of fat fraction, which indicate their connection between the parameters and fat fraction and could be referred for the following experiments on patient data.

**Table 3:** Experimental results on phantoms

Estimated parameter	Phantom		
	040GSE 0.5 dB region	040GSE 0.7 dB region	House-made oil phantom
Echo attenuation (dB/MHz/cm)	0.5132 ± 0.0367	0.7018 ± 0.0373	0.7563 ± 0.1605
Elasticity (kPa)	22.1111 ± 8.5971	17.8650 ± 5.1068	51.6250 ± 5.8750
Dispersion slope (m/s/Hz)	0.4821 ± 0.2612	0.4518 ± 0.3792	3.5391 ± 2.2279
Shear wave attenuation (Neper/m)	65.9084 ± 4.0722	74.8187 ± 18.3371	51.9126 ± 17.4872
Shear wave absorption (Neper/m)	53.6819 ± 1.4880	61.9683 ± 3.8338	38.4690 ± 5.1999

### Patient results

**Fig. 2:**
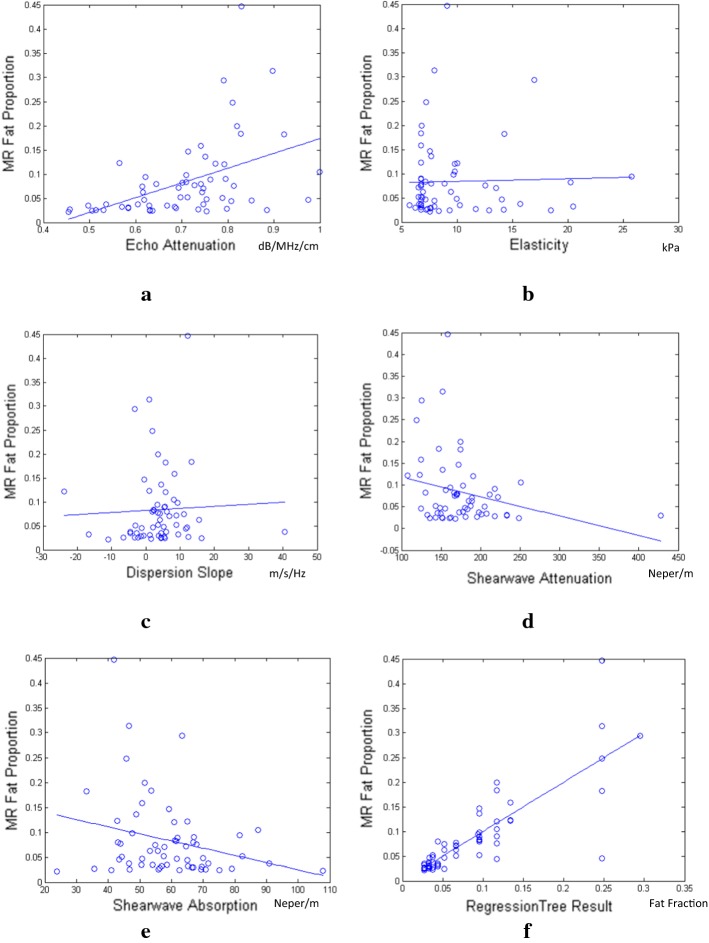
The experimental results of all 60 patient data denoting the relationship between the estimated parameters and the liver fat proportion from MR mDIXON quantification, which are **a** echo attenuation, **b** elasticity, **c** dispersion slope, **d** shear wave attenuation, **e** shear wave absorption and **f** model using the combination of all parameters

The result distributions of the estimated parameters and the learning-based model’s output for different fat proportion segments were demonstrated in the second part of Table 2. Figure 2 further demonstrates the correlation distributions of the results for all 60 patient data with the MR mDIXON quantification results. To quantitatively evaluate the different methods, the correlation coefficient (CC) values are applied between the estimation results and the liver fat proportion from MR mDIXON quantification [16]. The details of the related CC values are shown in Table 4. Among the individual parameter estimation, echo attenuation achieves the best performance. Compared with echo attenuation, shear wave absorption and shear wave attenuation are less effective. They demonstrate as negative correlation with liver fat fraction, which is consistent with the observation from the phantom results. Low-correlation coefficients with liver fat proportion are manifested for elasticity and viscosity, which denotes their limited capability on quantifying liver fat proportion individually. However, the performance significantly improves for the learning-based model by combining all the parameters together and the CC value achieves as high as 0.83. The detailed regression results of the model are further analyzed by comparing it with the MR mDIXON quantification results. The root mean square error is only 4.47% for liver fat proportion estimation, which achieves state-of-the-art for ultrasound liver fat quantification [3].

**Table 4:** The correlation coefficients between the estimation results and the liver fat proportion from MR mDIXON quantification

Methods	Correlation coefficients	*P* value
Echo attenuation	0.4594 (95% CI 0.2327 to 0.6388)	0.00022
Elasticity	0.0283 (95% CI -0.2273 to 0.2802)	0.83
Dispersion slope	0.0447 (95% CI -0.2116 to 0.2953)	0.73
Shear wave attenuation	− 0.2542 (95% CI − 0.4773 to − 0.0003)	0.050
Shear wave absorption	− 0.2599 (95% CI − 0.4820 to − 0.0064)	0.044
Model using the combination of all parameters	0.8305 (95% CI 0.7306 to 0.8956)	< 0.00001

**Fig. 3:**
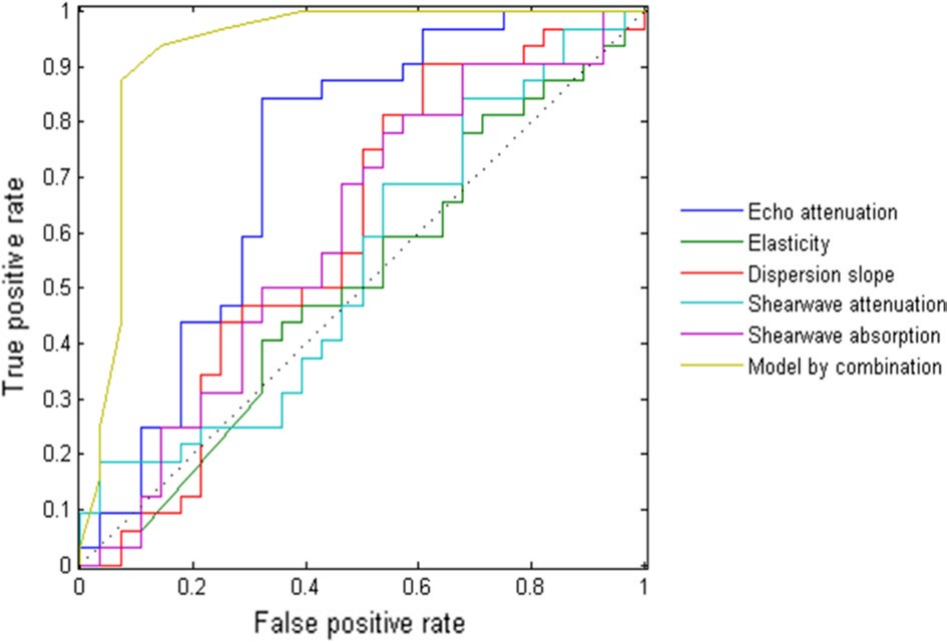
The receiver operating characteristic (ROC) curves of different methods for the discrimination of fatty liver disease

**Fig. 4:**
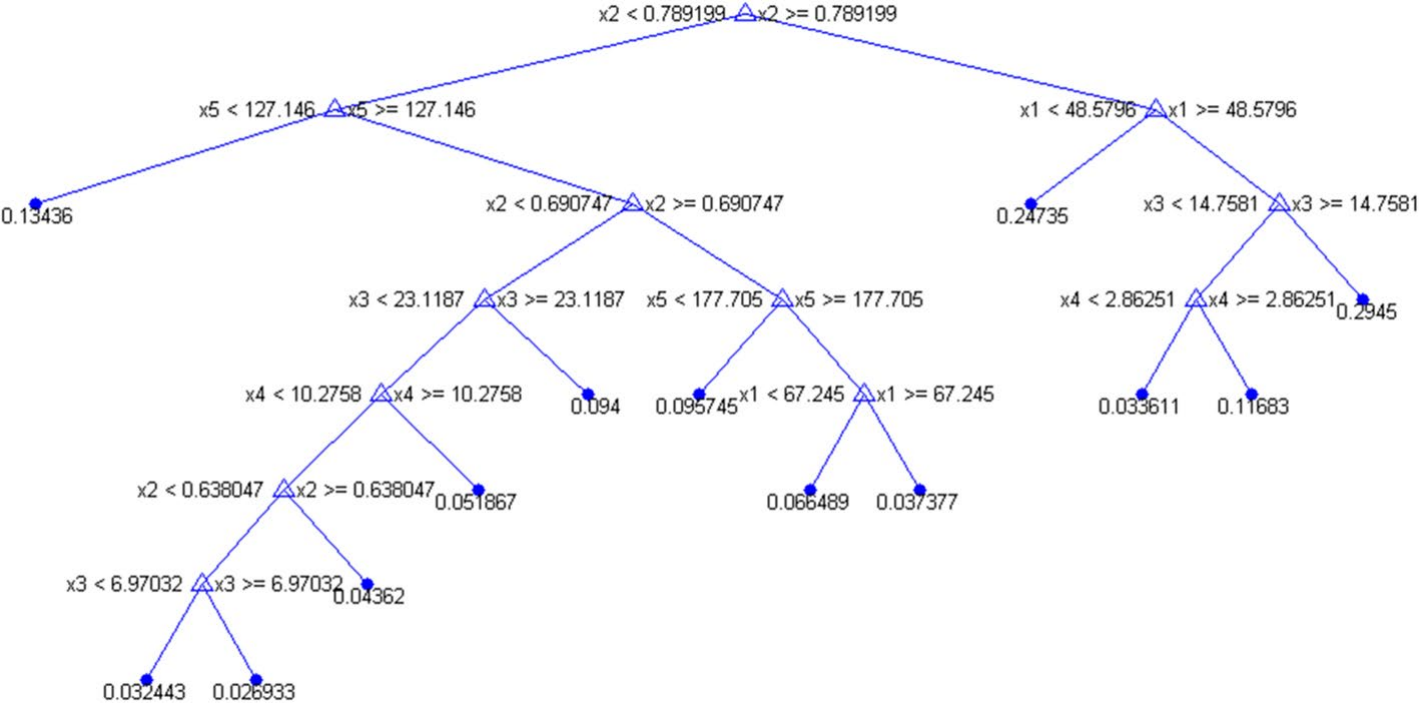
The details of the established regression tree model by combining all the parameters where ×1, ×2, ×3, ×4 and ×5 denote the parameter values of shear wave absorption, echo attenuation, elasticity, dispersion slope and shear wave attenuation, respectively

We further perform the statistical analysis to investigate the experimental results. Since the percentage values of liver fat proportion is the liver fat quantification results of MR mDIXON quantification, it is needed to apply a thresholding value as a certain percentage to identify the patient label for the discrimination of fatty liver disease to evaluate different methods. In our study, the thresholding value was chosen as 5% for the detailed evaluation, which is based on the previous clinical studies [1, 3]. Sensitivity (SEN), specificity (SPC), positive predictive value (PPV), negative predictive value (NPV), accuracy (ACC) and the area under the receiver operating characteristic (AUC) are the detailed evaluation criteria. The receiver operating characteristic (ROC) curves of different methods are demonstrated in Fig. 3. It can be observed that the result of the established model using all the estimated parameters outperforms the ones of individual parameters obviously. The optimal cut-off values from the ROC curves are determined based on Youden’s index [17] and based on them, the detailed statistical results are shown in Table 5. From the quantitative evaluation results, echo attenuation achieves the best performance among the individual parameters. This is consistent with the information from the structure of the established regression tree model in which echo attenuation plays a significant role as the fundamental node shown as in Fig. 4. For echo attenuation to discriminate fatty liver disease, the optimal cut-off value is 0.69 dB/MHz/cm in our study and it is consistent with the previously reported work [18]. Furthermore, Table 5 clearly manifests the significant improvement from the model by combining all the parameters. The accuracy as 76% for echo attenuation increased to 90% as for the established model. Since echo attenuation is comparable with the B-mode assessment by experienced doctors [18], the proposed learning-based model can help significantly increase the accuracy of the diagnosis of fatty liver disease for clinical practice. For the outputs of the established model, the optimal cut-off value is 6.6%. It is close to the selected 5% for the discrimination of fatty liver disease.

**Table 5:** The statistical analysis of the experimental results of different methods regarding the discrimination of fatty liver disease

Method	Statistical results with the corresponding optimal cut-off values from ROC curves for different methods					
	SEN (%)	SPC (%)	PPV (%)	NPV (%)	ACC (%)	AUC
Echo attenuation	84.38	67.86	75.00	79.17	76.67	0.73
Elasticity	78.13	32.14	56.82	56.25	56.67	0.51
Dispersion slope	90.63	39.29	63.04	78.57	66.67	0.60
Shear wave attenuation	84.38	32.14	58.70	64.29	60.00	0.54
Shear wave absorption	78.13	46.43	62.50	65.00	63.33	0.60
Model by using the combination of all parameters	87.50	92.86	93.33	86.67	90.00	0.93

To further validate the effectiveness of the proposed method, we randomly use a small proportion of the entire patient data as the independent testing data and the rest of the data is correspondingly used for the establishment of the machine-learning model. To observe the performances of the established models under various conditions of clinical data availability, the proportion for the independent testing data varies as 5%, 10% and 15%, respectively. The repeated cross-validation number for each value of the proportion is 10 to statistically guarantee the precise evaluation of the established model. The results of the validation are demonstrated in Table 6. It can be seen that the performance of the proposed method is stable as maintaining CC value around 0.8 with the liver fat quantification results of MR mDIXON quantification. Meanwhile, when the proportion of the independent testing data increases, the model’s performance manifests a slight decrease, which denotes the data size does impact the performance of the model since it is a data-based machine-learning model.

**Table 6:** The validation results of the proposed method by randomly using a proportion of the patient data as the independent testing data and using the rest of the patient data to establish the corresponding machine-learning model

Proportion of the independent testing data in the entire patient data (%)	Correlation coefficients between the model’s estimation results and the liver fat proportion from MR mDIXON quantification	P value
5	0.8249 (95% CI 0.7223 to 0.8920)	< 0.00001
10	0.8049 (95% CI 0.6925 to 0.8791)	< 0.00001
15	0.7816 (95% CI 0.7173 to 0.8326)	< 0.00001

## Discussion

Ultrasound imaging is widely utilized in clinical practice. However, the conventional way highly depends on the operation of physician even just for diagnosing the existence of fatty liver disease. It is subjective, qualitative and often leads to the problem of non-reproducibility and inaccuracy. A previous study [3] attempted to realize the liver fat quantification. However, its quantification approach still mainly depended on the appropriate acquisition of the hepatic-renal imaging plane, which remains operator-dependent. To solve such problem, our study explored the potential method to only use the objective, robust and quantitative parameters for liver fat quantification. It should be noted that by intention, any operator-dependent process is excluded in the quantification of fatty liver.

From the results of individual estimated parameters, it can be observed that the attenuation in the axis-beam direction is positively correlated with the results of MR mDIXON quantification and conversely, the parameters of shear wave energy dissipation are negatively correlated. This interesting phenomenon demonstrates the difference between longitudinal ultrasound signal and shear ultrasound signal even based on the same characteristic change for the material. It provides the physical evidence regarding the characteristics of shear wave signals for the future study.

It can be seen that from our study, the combination of all parameters establishes the foundation of the excellent performance of the learning-based model for liver fat quantification. First, these parameters should be able to be robustly estimated, which guarantees the accuracy of the following established model. Furthermore, these parameters are preferable to be supplemented for each other on signal information. For a generated ultrasound propagation phenomenon, the obtained longitudinal and shear signals include all the generated signals. When the estimated parameters represent the entire signal information for these two directions, the ultrasound representation of the target tissue can be thus considered as well defined. Meanwhile, to use a mechanical model to define a target tissue, elasticity and viscosity can well define the mechanical model. Based on all the considerations, the five parameters in our study are chosen and applied for the related processing of tissue characterization. Such set of parameters is considered as a “complete” signal set. Here, the “complete” means that once the parameter values in such parameter set for a target tissue is determined, the target tissue can be well characterized and in the high-dimensional space defined by the parameter set, the target tissue can be thus well discriminated with other objectives even if the performance of individual parameters is limited. Such idea has been preliminarily verified by our study. It provides an approach for the research not just for liver fat quantification, but furthermore for the goal of tissue characterization.

Based on the characteristics of relatively simple operation, low examination cost and high penetration rate of medical ultrasound imaging, the proposed model by combining all the estimated parameters demonstrates its high clinical value for liver fat quantification. Compared with MR mDIXON quantification and liver biopsy, it provides a relatively better method for liver fat quantification in clinical work. Limitations of the present study also need to be mentioned. Subjects in our study were enrolled in a single clinical center and the sample size was relatively small, which should be improved in the future multi-center study.

## Conclusions

Individual ultrasound and shear wave parameters demonstrate limited capability for liver fat quantification. However, by combining them together, the established learning-based model may achieve a good performance. It verifies the idea that in the high-dimensional space defined by the proposed ultrasound parameter set, the target tissue can be well defined and discriminated with other objectives. It provides an approach not just for liver fat quantification, but furthermore for the goal of ultrasound tissue characterization.

## Methods

### Ultrasound multiple parameter estimation

When transmitting an ultrasound push at a focus in the longitudinal direction (through the beam axis), the shear wave signal resulting from the generated acoustic radiation force can be observed through the shear direction at the focus using tracking-echo beams adjacent to this push beam. For one measurement of all the ultrasound parameters in our study, the designed specific ultrasound shear wave sequence consists of three ultrasound pushes and each push focuses at a different depth. The tracking-echo signals with the format as raw radiofrequency (RF) data are correspondingly obtained after each push. Based on the received signals from such single ultrasound sequence, the methods for simultaneously estimating all the ultrasound parameters in our study are correspondingly proposed to be performed, for which the main consideration is the robustness of estimation.

Conventional echo attenuation is directly estimated from the tracking-echo RF signals. A least-squares method is applied with the learning-based optimization for its parameters [18]. The power spectrum of the backscattered echo signals can be described as

1 $${\text{Sp}}\left( {f,z} \right) = G\left( {f,z} \right) \cdot A\left( {f,z} \right) \cdot B\left( f \right)$$

where ${\text{Sp}}$ is the power spectrum of the backscattered echo signal with $f$ as frequency and $z$ as the depth. $G$ represents the combined effects from the settings of system and beamforming. $A$ represents the total attenuation through the path from the transducer surface to the depth of interest, which is defined as $A\left( {f,z} \right) = \exp ( - 4\beta fz)$ where $\beta$ is the effective attenuation coefficient of the conventional echo. $B$ denotes the backscatter coefficient in the region of interest that is defined as $B\left( f \right) = bf^{n}$ where $b$ is a constant coefficient and $n$ represents the dependence with frequency. By obtaining the echo signal with the same setting for system and beamforming from a reference phantom, the ratio of the echo signal’s power spectrums from the target to that from the reference phantom at the same depth can be expressed as

2 $${\text{RS}}\left( {f,z} \right) = \frac{{B_{\text{tar}} \left( f \right)}}{{B_{\text{ref}} \left( f \right)}} \cdot \frac{{A_{\text{tar}} \left( {f,z} \right)}}{{A_{\text{ref}} \left( {f,z} \right)}} = \frac{{b_{\text{tar}} f^{{n_{\text{tar}} }} }}{{b_{\text{ref}} f^{{n_{\text{ref}} }} }}\exp \left( { - 4\left( {\beta_{\text{tar}} - \beta_{\text{ref}} } \right)fz} \right)$$

where the subscripts of ${\text{tar}}$ and ${\text{ref}}$ represent the target tissue and the reference phantom, respectively. The natural logarithm of both sides of Eq. 2 is performed and the equation can be further expressed as

3 $$\ln \left\{ {RS\left( {f,z} \right)} \right\} = \ln \frac{{b_{\text{tar}} }}{{b_{\text{ref}} }} + \left( {n_{\text{tar}} - n_{\text{ref}} } \right)\ln f - 4\left( {\beta_{\text{tar}} - \beta_{\text{ref}} } \right)fz$$

A least-squares fitting process can be thus applied over the band of frequencies that are contained in the echo signals by Eq. 3 based on the known acoustic properties of the reference phantom. The conventional echo attenuation can be thus obtained for the target tissue.

For the shear wave signals at certain depths, elasticity can be robustly estimated with the method by measuring the shear time-to-peak displacement [19, 20]. For shear wave signals, it can be proven that in nearly incompressible soft tissues such as liver parenchyma in our study,

4 $$E = 3\rho \cdot c_{\text{shearwave}}^{2}$$

where $E$ is the elasticity also known as Young’s modulus. $\rho$ is the liver density that is assumed as 1.0 g/cm^3^ [19] here. $c_{\text{shearwave}}^{{}}$ is the shear wave propagation speed. With tracking-echo RF signals, the tissue displacement caused by shear wave propagation can be estimated and manifest the location change of the peaks of shear wave displacements from different tracking beams. Therefore, the shear wave propagation speed can be obtained by equivalently measuring the peak propagation speed. The elasticity can be thus estimated based on Eq. 4.

Since the shear wave dispersion slope is positively correlated with the viscosity, the shear wave dispersion slope is practically and robustly estimated with the obtained shear wave signals to represent the related tissue characteristics of viscosity in our study [12, 13, 20, 21]. The shear wave velocity $v_{\text{shearwave}}$ can be defined as

5 $$v_{\text{shearwave}} \left( f \right) = \frac{2\pi f \cdot \Delta d}{\Delta \varphi }$$

where $f$ is the frequency component. $\Delta d$ is the distance between two adjacent tracking beams for which $\Delta \varphi$ denotes the corresponding phase shift due to the stimulated shear wave propagation. It is noted that no rheological model is assumed in our study and only a linear model is applied for the shear wave dispersion estimation. Based on Eq. 5, the shear wave dispersion slope ${\text{SDS}}$ can be thus defined as

6 $${\text{SDS}} = \frac{{\partial v_{\text{shearwave}} \left( f \right)}}{\partial f}$$

for which the practical estimation from Eq. 6 is to calculate the slope of a linear fitting of shear wave velocities versus different frequency components with the range from 50 to 300 Hz.

Based on the shear wave signals from all the locations of tracking-echo beams, shear wave attenuation can be robustly estimated with a model-free method [14]. The maximum values of shear wave displacements are estimated for each tracking-echo beam. The ratios between these maxima and the one from the first tracking-echo beam are further calculated. These ratios are assumed as exponentially depending on the propagation distance of tracking-echo beams that can be written as

7 $${\text{MDR}}_{i} = e^{{ - \alpha \cdot d_{i} }}$$

where ${\text{MDR}}_{i}$ denotes the ratio of maximum shear wave displacements between the *i*th and the first tracking-echo beams. $d_{i}$ denotes the distance between the *i*th and the first tracking-echo beams. The estimation of shear wave attenuation is realized by calculating the slope α from the fitting of the Eq. 7. It is noted that the estimation of shear wave attenuation was performed only in the near region of every push focus in our study. The diffraction related to the acoustic radiation force [22, 23] could be thus negligible to some extent for the estimation.

It is hypothesized in our study that the energy dissipation in tissue could be an important bio-marker for liver lipid content. Therefore, the estimation of shear wave absorption is further introduced for liver fat quantification and it emphasizes the energy that is absorbed and transferred for the generation of shear wave phenomenon. Theoretically, for shear wave signals, it can be proven [24–26] that

8 $$\frac{{\partial^{2} S_{x} }}{{\partial t^{2} }} - \left( {c_{\text{shearwave}}^{2} + \gamma \frac{\partial }{\partial t}} \right)\Delta_{lp} S_{x} = F_{x}$$

where $x$ represents the beam axis. $t$ is time. $S_{x}$ denotes the shear wave displacement along the $x$ direction. $c_{\text{shearwave}}^{{}}$ is the shear wave propagation speed. $\gamma$ denotes the shear viscosity. $\Delta_{\text{lp}}$ denotes the Laplacian operator. $F_{x}$ represents the radiation force that stimulates the shear wave phenomenon. Based on Eq. 8 and with the further assumption of pulsing modulation, it can be theoretically derived [24–26] that

9 $$S_{{x_{\text{focus}} ,\text{max} }} = \frac{{\sqrt \pi \sigma a^{2} t_{0} I_{0} }}{8c\rho \gamma } \cdot e^{{ - 2\sigma \cdot x_{\text{focus}} }} \propto I_{0} \cdot e^{{ - 2\sigma \cdot x_{\text{focus}} }}$$

where $S_{{x_{\text{focus}} ,\text{max} }}$ denotes the maximum displacement of the generated shear wave at the focus depth $x_{\text{focus}}$. $a$ is the aperture size. $t_{0}$ denotes the initial time. $c$ is the ultrasound speed that could be assumed as 1540 m/s. $\rho$ is the liver density assumed as 1.0 g/cm^3^ here [19]. $\sigma$ denotes the shear wave absorption coefficient. $I_{0}$ is the acoustic intensity of the initial ultrasound wave on the beam axis that is defined as

10 $$I_{0} = \frac{{P_{0}^{2} }}{2c\rho }$$

where $P_{0}^{{}}$ represents the initial acoustic pressure.

For the details about the related theory of shear wave absorption coefficient, the work by Rudenko et al. [24] was well performed for the related theoretical derivations. Based on them, we propose an approach to practically realize the estimation of shear wave absorption coefficient. It can be seen that according to Eq. 9, $S_{{x_{\text{focus}} ,\text{max} }}$, $I_{0}$ and $x_{\text{focus}}$ are the only parameters that require to know their values for the practical estimation of shear wave absorption coefficient $\sigma$. Therefore, the approach for shear wave absorption estimation was to first obtain the maximum displacements of the generated shear waves focused at the three focus depths in our designed specific ultrasound shear wave sequence. Then, for every acoustic push, the applied voltage $V_{0}^{{}}$ on the transducer elements was recorded since $P_{0}^{{}}$ could be considered as proportional to the applied voltage $V_{0}^{{}}$ of the transducer elements. Thus $I_{0}$ based on Eq. 10 could be considered as proportional to the value of $\frac{{V_{0}^{2} }}{2c\rho }$, which is utilized as the practical value to compensate the difference for the initial intensities $I_{0}$ of the three different acoustic pushes. Since the ultrasound system is exactly the same one for different acoustic pushes, this approximation would not affect the shear wave absorption coefficient estimation at all. After compensating the initial intensities $I_{0}$ and obtaining the values as $\frac{{S_{{x_{\text{focus}} ,\text{max} }} }}{{I_{0} }}$, a curve fitting was simply performed with an exponential form in which the focus depth was the variable. The shear wave absorption coefficient $\sigma$ can be directly and conveniently calculated from this fitting.

### Model by using the combination of all parameters

Each of the above estimated parameters may represent some aspect of the physical characteristics of target tissue (as liver fat in our study). Therefore in our study, it was hypothesized that by simultaneously considering the entire set of these robustly estimated parameters, the target tissue should be able to be better represented and identified compared with using one individual parameter. In other words, the target tissue could be well characterized in the high-dimensional space defined by all the estimated parameters. To verify this hypothesis, a machine-learning method was applied in our study to establish the model in such high-dimensional space. However, to avoid the over-fitting for the learning-based model since the sample number in our study was as small as 60, we should select the model that is relatively simple compared with the state-of-the-art ones. Based on such consideration, the regression tree was applied as the learning-based model in our study [27]. Figure 4 demonstrated the details of the established regression tree in which ×1, ×2, ×3, ×4 and ×5 denote the parameter values of shear wave absorption, echo attenuation, elasticity, dispersion slope and shear wave attenuation, respectively. This model is for the purpose to investigate the potential connections for the physical characteristics represented by the different estimated parameters. The hierarchical structure of the model approximates the non-linearity in the parameter space while maintaining the low-computational complexity that is specifically appropriate for clinical practice.

## Data Availability

The datasets used and/or analyzed during the current study are available from the corresponding author upon reasonable request.

## Abbreviations

CC: correlation coefficient
AUC: area under the receiver operating characteristic
NASH: non-alcoholic steatohepatitis
NAFL: non-alcoholic fatty liver
MRI-PDFF: magnetic resonance imaging based proton density fat fraction
BSC: backscatter coefficient
BMI: body mass index
RF: radiofrequency
SEN: sensitivity
SPC: specificity
PPV: positive predictive value
NPV: negative predictive value
ACC: accuracy
ROC: receiver operating characteristic
